# Role of tPA in Corticosterone-Induced Apoptosis of Mouse Mural Granulosa and Oviductal Epithelial Cells

**DOI:** 10.3390/cells12030455

**Published:** 2023-01-31

**Authors:** Qi Hua, Hao Cheng, Yong-Qing Yang, Jin-Song An, Min Zhang, Shuai Gong, Ming-Jiu Luo, Jing-He Tan

**Affiliations:** Shandong Provincial Key Laboratory of Animal Biotechnology and Disease Control and Prevention, College of Animal Science and Veterinary Medicine, Shandong Agricultural University, Taian 271018, China

**Keywords:** apoptosis, glucocorticoids, mural granulosa cell, oviductal epithelial cell, tissue plasminogen activator

## Abstract

Although studies indicate that female stress-increased secretion of glucocorticoids impairs oocyte competence and embryo development, by inducing apoptosis of ovarian and oviductal cells, respectively, the mechanisms by which glucocorticoids induce apoptosis of ovarian and oviductal cells are largely unclear. Tissue plasminogen activator (tPA) has been involved in apoptosis of different cell types. However, while some studies indicate that tPA is proapoptotic, others demonstrate its antiapoptotic effects. This study has explored the role and action mechanisms of tPA in corticosterone-induced apoptosis of mouse mural granulosa cells (MGCs) and oviductal epithelial cells (OECs). The results demonstrate that culture with corticosterone significantly increased apoptosis, while decreasing levels of tPA (Plat) mRNA and tPA protein in both MGCs and OECs. Culture with tPA ameliorated corticosterone-induced apoptosis of MGCs and OECs. Furthermore, while tPA protected MGCs from corticosterone-induced apoptosis by interacting with low-density lipoprotein receptor-related protein 1 (LRP1), it protected OECs from the apoptosis by acting on Annexin 2 (ANXA2). In conclusion, tPA is antiapoptotic in both MGCs and OECs, and it protects MGCs and OECs from corticosterone-induced apoptosis by interacting with LRP1 and ANXA2, respectively, suggesting that tPA may use different receptors to inhibit apoptosis in different cell types.

## 1. Introduction

Although studies have shown that stress can impair female reproduction, the mechanisms by which various stressors harm the oocyte and the embryo are largely unclear. Recent studies indicate that restraint-stress in female mice impaired oocyte competence [1] and preimplantation embryo development [2], with increased secretion of corticotrophin-releasing hormone (CRH) and/or glucocorticoid hormones, which damages oocytes and embryos by inducing apoptosis of ovarian cells [3,4,5,6,7] and oviductal cells [8], respectively. However, the mechanisms by which stress hormones induce apoptosis of ovarian and oviductal cells are largely unclear. Furthermore, while culture with corticosterone induced apoptosis in mouse ovarian mural granulosa cells (MGCs) by activating the TNF-α system [5], it induced apoptosis of mouse oviduct epithelial cells (OECs) independent of the TNF-α system [9]. Thus, whether glucocorticoids trigger apoptosis of MGCs and OECs through different signaling pathways is worth exploring. 

While it is known that tissue-type plasminogen activator (tPA) plays an important role in the vascular and central nervous system, its production by many other cell types suggests its functions in other systems as well [10]. For instance, tPA has been involved in apoptosis of different cell types. However, while some studies indicate that tPA is proapoptotic [11,12], others demonstrate its antiapoptotic effects [13,14]. The mechanisms by which tPA mediates such opposite functions remain unclear although several hypotheses have been proposed [15]. Furthermore, tPA is expressed in the ovary, including granulosa cells [16], and in the oviducts of mammals [17,18]. Thus, the role of tPA in glucocorticoid-induced apoptosis of MGCs and OECs must be studied, to understand the mechanisms by which stress hormones damage oocytes and embryos, and to reveal how it can mediate the directly opposite pro- and anti-apoptotic functions. 

Plasma membrane receptors for tPA include the low-density lipoprotein receptor-related protein (LRP) and the annexin A2 (ANXA2) [19,20,21]. However, most studies published so far on the roles of these receptors concluded that they bind tPA and plasminogen, and concentrate their proteolytic activity on the cell surface by promoting the interaction of these ligand proteins [22]. Although it is of great importance to understand the signaling pathways by which tPA regulates cell apoptosis, reports of tPA regulating cell apoptosis via its receptors are very limited; we could find only one paper reporting that the antiapoptotic effect of tPA was independent of its protease activity but required its membrane receptor LRP1 [13]. 

Thus, the objectives of the present study were to find out whether tPA is involved in regulation of the glucocorticoid-induced apoptosis in mouse MGCs and OECs; whether it uses its plasma membrane receptors to regulate the apoptosis; and whether it uses the same or different receptors (or signaling pathways) in different cells. We aimed to understand the mechanisms by which stress hormones damage oocytes and embryos and the signaling pathways that tPA uses to inhibit or promote cell apoptosis. 

## 2. Materials and Methods

Unless otherwise pointed out, all the chemicals and reagents used in this study were bought from Sigma-Aldrich (St. Louis, MO, USA).

### 2.1. Animals and Recovery of Ovaries and Oviducts

Animal care and handling were carried out in accordance with the guidelines authorized by the Shandong Agricultural University Animal Care and Use Committee, P. R. China (Permit number: SDAUA-2019-004). Mice of the Kunming strain, which were originally derived from ICR (CD-1) mice, were used in this study. The mice were kept in rooms under a photoperiod of 14-h light/ 10 h darkness, with lights off at 20:00 h. Female mice (8–12 weeks after birth) were injected with eCG (10 IU/mouse), and at 48 h after eCG injection, the mice were sacrificed to recover ovaries and oviducts.

### 2.2. Culture of MGCs and OECs

The large follicles on the ovary were ruptured in an M2 medium to release oocytes, and the MGCs sheets released into the M2 medium at the follicles’ puncture-point were recovered for culture. The MGCs sheets recovered were centrifuged for 5 min at 200× *g*, and then digested for 3 min with 0.25% trypsin at 37.5 °C. The oviducts recovered were cut into segments, and each segment was squeezed using forceps to extrude the oviduct epithelial tissue. The tissue blocks were collected and digested with 0.25% trypsin at 37 °C for 40 min. The MGCs and OECs thus obtained were washed by centrifugation at 200 g for 5 min in DMEM/F12 (Gibco) supplemented with 10% (*v*/*v*) fetal calf serum (Gibco) and 0.5% (*v*/*v*) penicillin–streptomycin solution (Gibco). After the cell concentration was adjusted to 1 × 10^5^ cells /mL, 1 mL (MGCs) or 0.5 mL (OECs) of the final suspension was added to each well of a 6-well (MGCs) or 12-well (OECs) culture plate, and incubated at 37.5 °C in a humidified atmosphere of 5% CO_2_ in air. After that, the culture medium was renewed at 24-h intervals. When the OECs grew to about 80–90% of confluence, the cells in each well were digested with trypsin, resuspended in 2 mL DMEM/F12, and sub-cultured in two wells (1 mL per well) of a 12-well culture plate. When growing to 70–80% of confluence, the MGCs and the sub-cultured OECs were cultured for 24 h and 48 h, respectively, in the presence of corticosterone with or without tPA. 

### 2.3. Hoechst 33342 Staining for Assessment of Cell Apoptosis

Following corticosterone and/or tPA treatment, the primary-cultured MGCs and sub-cultured OECs were stained in situ in culture wells using 10 μg/mL of Hoechst 33342 for 5 min in the dark. After staining, the cells were examined with a Leica DMLB fluorescence microscope (400×), and 6–8 fields were randomly examined in each well. While the apoptotic cells showed pyknotic nuclei full of heavily stained heterochromatin with bright fluorescence, healthy cells showed normal nuclei with sparse heterochromatin spots. Eighty to one hundred cells were counted in each field to calculate the percentages of apoptotic cells. 

### 2.4. Flow Cytometry Assessment of Cell Apoptosis

We used a BD Pharmingen FITC Annexin V Apoptosis Detection Kit (BD Biosciences, 0027279) for the assay. The MGCs/OECs were first stained with Annexin V- FITC/propidium iodide (PI) staining and then measured by flow cytometry. Briefly, we washed MGCs/OECs in PBS, and digested them at 37 °C with 0.25% trypsin for 3 to 5 min. Then, we added DMEM/F12 medium containing 10% fetal bovine serum to neutralize the residual trypsin. Finally, we resuspended cells of each sample in 100 μL binding buffer, containing 5 μL Annexin V-FITC and 5 μL PI, and incubated for 15 min in the dark before analysis using a LSRFortessa (BD, Franklin Lakes, NJ, USA). 

### 2.5. Real-Time PCR

To isolate total RNA, we removed the spent medium from wells culturing MGCs/OECs, and added 1 mL Trizol reagent to each well. We pipetted cells for 5 min to facilitate lysis. Then we resuspended the RNA in DEPC-dH_2_O before RNase-free DNase 1 (Takara Biotechniques, Dalian, China) digestion. We quantified the RNA spectroscopically at 260 nm. To assess RNA purity and integrity, we determined the ratio of A260:A280 (1.8–2.0) and carried out electrophoresis in 1% agarose.

We conducted reverse transcription with a total volume of 20 μL containing Superscript III Reverse Transcriptase (Invitrogen Australia Pty. Ltd., Mulgrave, Australia). We first mixed 2 μL of RNA sample, 1 μL Oligo dT18 (Takara), and 10 μL DEPC-dH_2_O in a reaction tube, and incubated the mixture at 65 °C for 10 min in a PCR instrument (Thermo Scientific, Hudson, NH, USA). We then cooled the reaction tube for 2 min on ice before a brief centrifugation (200× *g*, 4 °C). We finally added 5× reverse transcription buffer (4 μL), RNase inhibitor (0.5 μL), dNTP (2 μL), and Superscript III Reverse Transcriptase (0.5 μL) to the reaction tube, and incubated the mixture at 42 °C for 1 h and at 70 °C for 15 min, before storing at −80 °C until use.

Gene-specific primers used are shown in Table 1. We used a Mx3005P Real-Time PCR System (Stratagene, Valencia, CA, USA) to conduct mRNA quantification using a 10-μL reaction volume containing 1 μL of cDNA, 5 μL of 2 × SYBR Green Master Mix (Stratagene), 0.15 μL of 500 × diluted reference dye, 3.25 μL of RNase-free water, and 0.3 μL each of forward and reverse primers (10 μM). We used the following cycle amplification conditions: (1) denaturation at 95 °C for 10 min, and (2) 40 cycles at 95 °C for 5 s, and at 58 °C (Bax and Bcl-2), 62 °C (Plat), 57 °C (Lrp1) or 56 °C (Anxa2) for 20 s. We normalized expression of all these genes to internal control (Gapdh), with all values expressed relative to the calibrator samples using the 2^–(ΔΔCT)^ method.

The Normfinder (https://www.moma.dk/normfinder-software/, accessed on 30 November 2022), an algorithm-based tool, was adopted to identify the most stable genes among the seven widely used reference genes. The samples used for the analysis were obtained from 10 different OECs subcultures. Our calculation using Normfinder indicated that the ranking of expression stability in the genes analyzed was Gapdh > Actb > B2m > Ppib > H2a > Hprt1 > Rplpo. Thus, Gapdh was used as a stable internal reference gene in our PCR assays. For MGCs, we also used Gapdh for internal reference as previously reported [23,24]. 

### 2.6. Enzyme-Linked Immunosorbent Assay (ELISA) for tPA Protein

The tPA protein in a conditioned medium (CM), conditioned by MGCs and OECs, was detected by ELISA using a BCA Protein Assay Kit (CW0014S, CoWin Biosciences, Beijing, China) and Mouse Tissue Polypeptide Antigen, TPA ELISA Kit (YM-S3252, Shanghai yuan Mu Biotechnology Co., Ltd., Shanghai, China). The primary MGCs and sub-cultured OECs were cultured for 24 h and 48 h, respectively, in the presence of corticosterone before collection of the CM. The CM recovered was subjected to BCA protein quantification and ELISA assays. The procedures for the assays were carried out exactly following the manufacturer’s instructions. 

### 2.7. Cell Transfection with siRNAs

The targeting siRNAs and negative control siRNAs were designed and synthesized by RiboBio (Guangzhou, China). The sense strands of targeting siRNAs for Lpr1 included: (1) Lpr1 siRNA-1 (5′-GCA GCG AGC CAA CAA GTAT-3′), Lpr1 siRNA-2 (5′-CCA ACT ACA CAC TGC TTA A-3′) and Lpr1 siRNA-3 (5′-GCA GGT TGT TAG TCA GCA A -3′); (2) those for Anxa2 included Anxa2 siRNA-1 (5′-CCT GAC AAA CCG CAG CAA T-3′), Anxa2 siRNA-2 (5′-GGA AAT GTA CAA GAC TGA T-3′) and Anxa2 siRNA-3 (5′-GCA AGT CCC TGT ACT ACT A-3′); and (3) siR-Ribo^TM^ for negative control. 

We conducted transfection with 100 nM siRNAs using Lipofectamine RNAiMAX reagent (Invitrogen/Life Technologies). When the MGCs and OEC subcultures attained 50% of confluence, we replaced the spent medium in the wells with 900 μL of fresh DMEM/F12, and transfected the cells using the forward transfection method. We prepared the transfection complex as follows: (1) 5 μL of a 20 μM solution of each siRNA was diluted in 45 μL of Opti-MEM medium (Invitrogen); (2) mixed with 3 μL of lipofectamine RNAiMAX reagent (Invitrogen) diluted in 47 μL of Opti-MEM medium. We then incubated the transfection complex for 5 min at room temperature, added it to the wells with cells, and incubated the cells for 48 h at 37 °C under a humidified atmosphere of 5% CO_2_ in air.

### 2.8. Data Analysis

Each treatment contained at least three replicates. The percentage data were arcsine-transformed before analysis. We analyzed data using one-way ANOVA when each measure contained more than two groups, and used the independent t-test when each measure contained only two groups. We performed a Duncan multiple comparison test to determine differences during ANOVA. We used the Statistics Package for Social Science (SPSS 20, SPSS, Chicago, IL, USA) to conduct data analysis. All the data are expressed as mean ± SEM, and the differences were considered significant only when the P value was less than 0.05.

## 3. Results

### 3.1. Culture with Corticosterone Increased Apoptosis of MGCs and OECs

**Figure 1:**
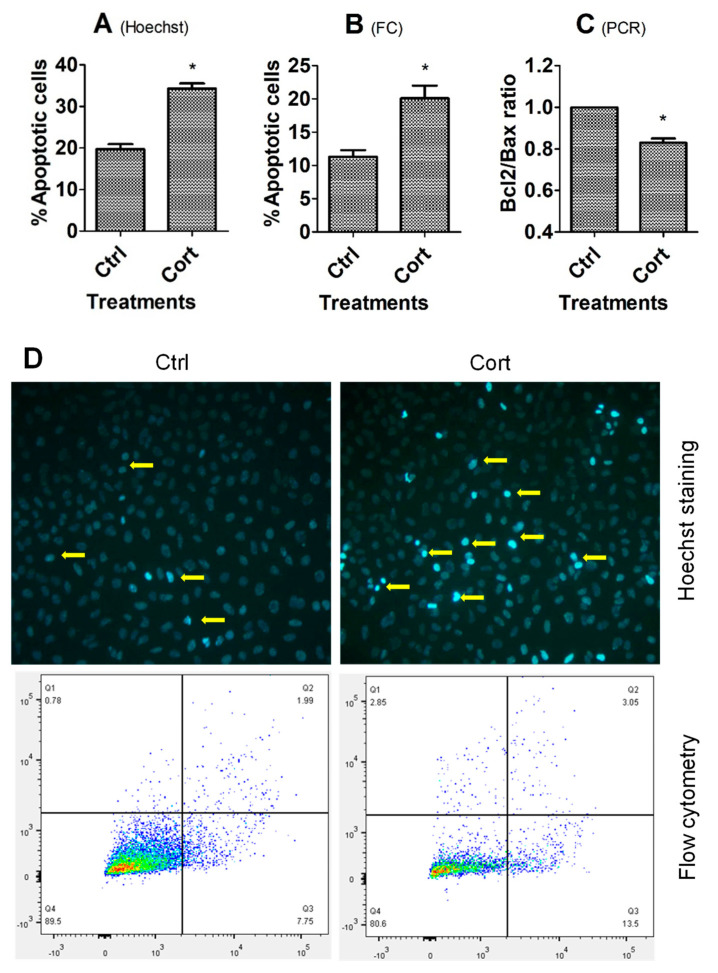
Effects of corticosterone treatment on apoptosis of MGCs. Graphs (**A**–**C**) show percentages of apoptotic cells revealed by Hoechst staining or flow cytometry (FC), and the ratio of Bcl2/Bax measured by RT-PCR, respectively, between untreated control (Ctrl) and corticosterone (Cort) treated cells. Each treatment was repeated 3 times with each replicate including cells from 2 wells of a 6-well plate from 12 mice. * Indicates significant difference (*p* < 0.05) from controls. In graph (**B**), the percentages of apoptotic cells included both early and late apoptotic cells. In graph (**C**), the ratio of Bcl2/Bax mRNA in Ctrl cells was set to one and the ratio in Cort-treated cells was expressed relative to it. Panel (**D**) shows images and the flow cytometry graphs of MGCs in the Ctrl or Cort groups. The MGC images were obtained under a fluorescence microscope after Hoechst staining. Original magnification was 400×. Arrows indicate representative apoptotic cells with pyknotic nuclei heavily stained by Hoechst 33342. The flow cytometry graphs were obtained following annexin-V and PI staining. The healthy, early and late apoptotic cells are located in the Q4, Q3 and Q2 area, respectively. The Q1 area contains necrotic cells and mechanically damaged cell debris.

**Figure 2:**
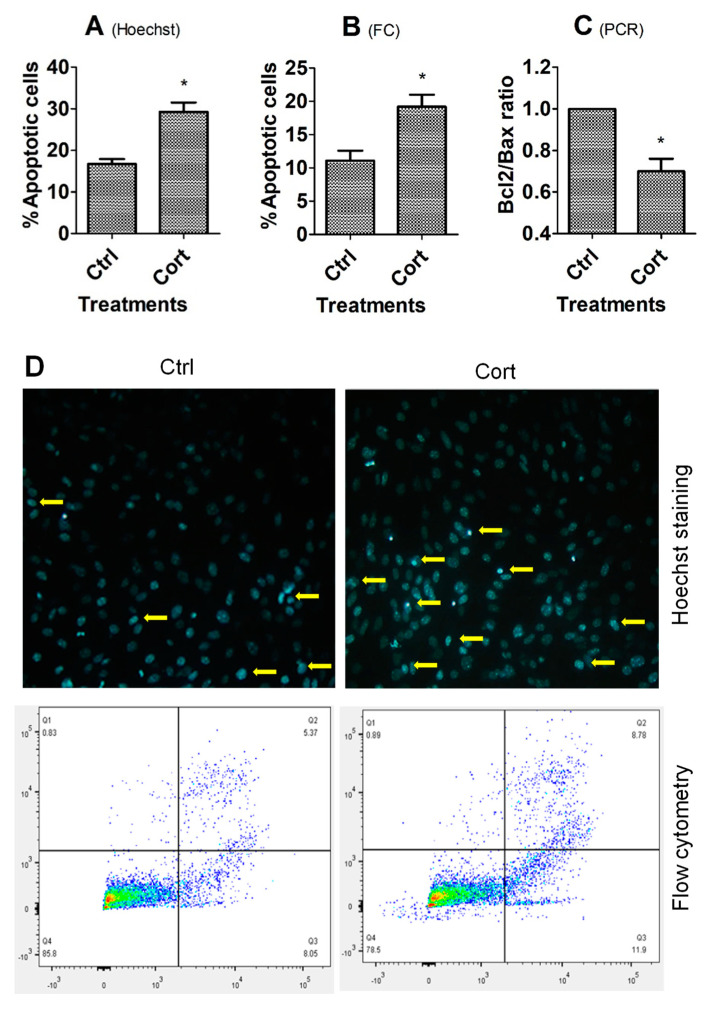
Effects of corticosterone treatment on apoptosis of OECs. Graphs (**A**–**C**) show percentages of apoptotic cells revealed by Hoechst staining or flow cytometry (FC), and the ratio of Bcl2/Bax measured by RT-PCR, respectively, between untreated control (Ctrl) and corticosterone (Cort) treated cells. Each treatment was repeated 3 times, with each replicate including cells from 2 wells of a 12-well plate from 4 mice. * Indicates significant difference (*p* < 0.05) from controls. In graph B, the percentages of apoptotic cells included both early and late apoptotic cells. In graph (**C**), the ratio of Bcl2/Bax mRNA in Ctrl cells was set to one and the ratio in Cort-treated cells was expressed relative to it. Panel (**D**) shows images and the flow cytometry graphs of OECs in the Ctrl or Cort groups. The OEC images were obtained under a fluorescence microscope after Hoechst staining. Original magnification was 400×. Arrows indicate representative apoptotic cells with pyknotic nuclei heavily stained by Hoechst 33342. The flow cytometry graphs were obtained following annexin-V and PI staining. The healthy, early and late apoptotic are located in the Q4, Q3 and Q2 area, respectively. The Q1 area contains necrotic cells and mechanically damaged cell debris.

When MGCs and sub-cultured OECs grew to 70–80% of confluence, the spent medium was replaced with freshly prepared medium without (control), or with 10^−5^ M of corticosterone, and then the cells were cultured for 24 h (MGCs) or 48 h (OECs) before sampling for Hoechst staining, flow cytometry, or RT-PCR analysis. In both MGCs (Figure 1) and OECs (Figure 2), while percentages of apoptotic cells revealed by either Hoechst staining or flow cytometry were higher, the Bcl2/Bax ratio was significantly lower in corticosterone-treated cells than in control cells. 

### 3.2. Culture with Corticosterone Decreased Expression of tPA Gene in MGCs and OECs

**Figure 3:**
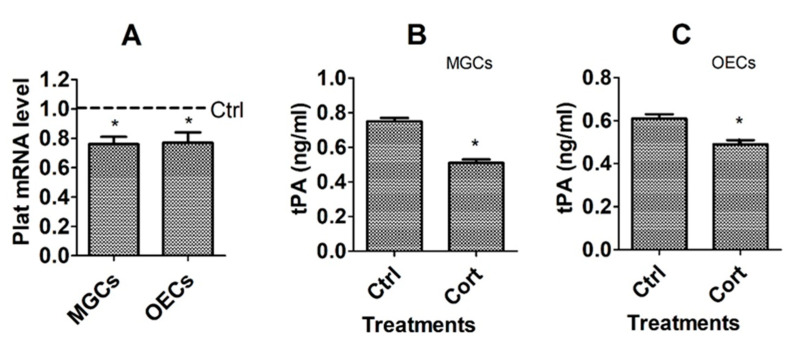
Effects of corticosterone treatment on mRNA and protein levels of tPA in MGCs and OECs. Graph (**A**) shows mRNA level of the Plat gene revealed by RT-qPCR. Each treatment was repeated 3 times with each replicate, including cells from 2 wells of a 6-well plate from 12 mice (MGCs) or 2 wells of a 12-well plate from 4 mice (OECs). The mRNA level in control (Ctrl) cells was set to one (dotted line) and the level in corticosterone-treated cells was expressed relative to it. Graphs (**B**,**C**) show levels of tPA protein in CM conditioned for 24 h by MGCs and OECs, respectively, in the presence of corticosterone, as revealed by ELISA. Each treatment was repeated 3 times, with each replicate including CM from 2 wells of a 6-well plate from 12 mice (MGCs), or 2 wells of a 12-well plate from 4 mice (OECs). * Indicates significant difference (*p* < 0.05) from Ctrl cells.

To observe the effects of corticosterone treatment on tPA expression, mRNA levels of the Plat (Plasminogen Activator, Tissue Type) gene, which encodes tPA, were assayed by RT-qPCR, and concentrations of tPA in conditioned medium were assessed by ELISA following culture of MGCs and OECs with or without corticosterone. The results showed that corticosterone treatment significantly decreased mRNA levels of the Plat gene (Figure 3A) and the level of tPA protein in both MGCs (Figure 3B) and OECs (Figure 3C). Thus, treatment with corticosterone significantly decreased expression of tPA in both MGCs and OECs, suggesting that corticosterone induced apoptosis in these cells by downregulating tPA expression. 

### 3.3. Culture with tPA Ameliorated Corticosterone-Induced Apoptosis of MGCs and OECs

**Figure 4:**
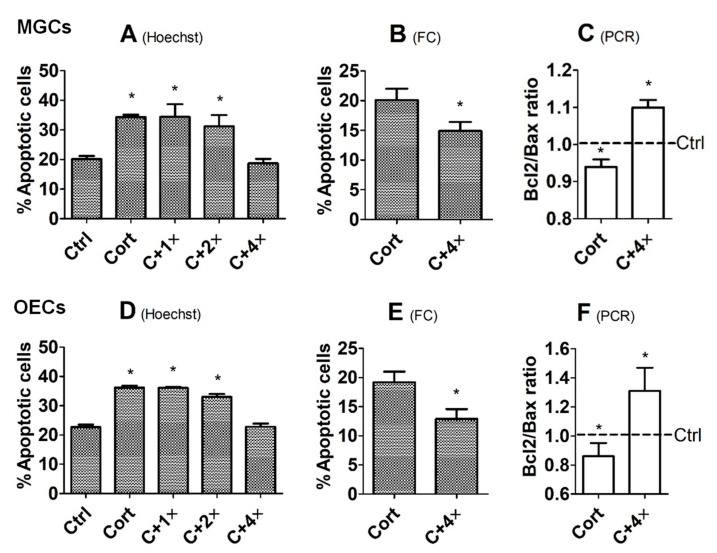
Effects of culture with tPA on corticosterone-induced apoptosis of mouse MGCs and OECs. While graphs (**A**–**C**) show percentages of apoptotic cells revealed by Hoechst staining or flow cytometry (FC) and the ratio of Bcl2/Bax measured by RT-PCR, respectively, in MGCs, graphs (**D**–**F**) show the same results in OECs. Each treatment was repeated 3 times, with each replicate including cells from 2 wells of a 6-well plate from 12 mice (MGCs) or from 2 wells of a 12-well plate from 4 mice (OECs). Ctrl, control; Cort, corticosterone (10^−5^ M); C + 1×, Cort + 1 × 10^−8^ M tPA; C + 2×, Cort + 2 × 10^−8^ M tPA; C + 4×, Cort + 4 × 10^−8^ M tPA. * Indicates significant difference (*p* < 0.05) from Ctrl or Cort cells. In graphs (**B**,**E**), the percentages of apoptotic cells included both early and late apoptotic cells. In graphs (**C**,**F**), the ratio of Bcl2/Bax mRNA in Ctrl cells was set to one (dotted line) and the ratio in Cort or C + 4× treated cells was expressed relative to it.

To verify that corticosterone triggers apoptosis by downregulating tPA expression, MGCs and OECs were cultured in the presence of corticosterone with or without various concentrations of tPA before assessment of apoptosis. Our Hoechst staining indicated that in the presence of corticosterone, percentages of apoptotic cells decreased significantly to the same level as observed in control cells cultured without corticosterone when the tPA concentration increased to 4 × 10^−8^ M in both cell types (Figure 4A,D). Similarly, our flow cytometry showed that culture with 4 × 10^−8^ M of tPA significantly reduced the apoptotic percentage of corticosterone-treated MGCs and OECs (Figure 4B,E). Furthermore, our RT-PCR also demonstrated that culture with 4 × 10^−8^ M of tPA significantly increased the Bcl2/Bax ratio in both MGCs and OECs (Figure 4C,F). 

### 3.4. Effects of Knocking Down the Lrp1 Gene on Corticosterone-Induced Apoptosis of MGCs and OECs

**Figure 5:**
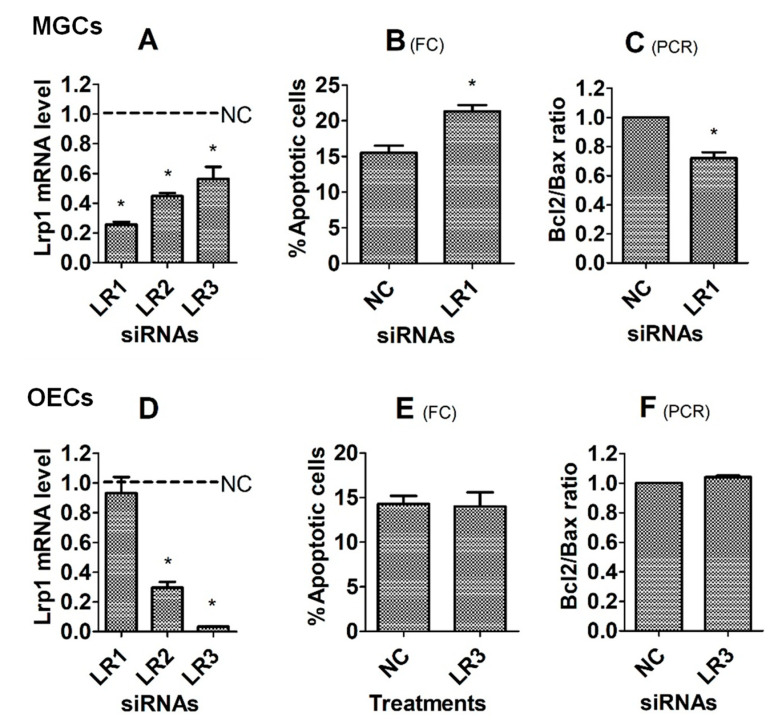
Effects of knocking down Lrp1 on corticosterone-induced apoptosis of MGCs and OECs. Graphs (**A**,**D**), (**B**,**E**) and (**C**,**F**) show Lrp1 mRNA level, percentage of apoptotic cells, and the ratio of Bcl2/Bax mRNA, respectively, after MGCs or OECs were transfected with negative control (NC), Lrp1 siRNA-1 (LR1), -2 (LR2) or -3 (LR3). While the mRNA level was revealed by RT-PCR, the percentage of apoptotic cells was measured by flow cytometry (FC). Each treatment was repeated 3 times with each replicate including cells from 2 wells of a 6-well plate from 12 mice (MGCs) or from 4 mice (OECs). * Indicates significant difference (*p* < 0.05) from NC cells. In graphs (**B**,**E**), the percentages of apoptotic cells included both early and late apoptotic cells. In graphs (**C**,**F**), the ratio of Bcl2/Bax mRNA in NC cells was set to one and that in LR1 or LR3 cells was expressed relative to it.

To further verify that tPA protects cells from apoptosis by interacting with its receptors, Lrp1 was knocked down by RNA interference before assays for corticosterone-induced apoptosis. To evaluate the silencing efficiency of different siRNA sequences, levels of Lrp1 mRNA were measured by RT-PCR immediately following transfection of MGCs or OECs with negative control, Lrp1 siRNA-1, -2 and -3. While the Lrp1 mRNA level decreased to the lowest in MGCs transfected with Lrp1 siRNA-1 (Figure 5A), it decreased to the lowest in OECs transfected with Lrp1 siRNA-3 (Figure 5D). Thus, Lrp1 siRNA-1 and -3 were used for MGCs and OECs, respectively. To test the effects of genes knocking down on corticosterone-induced apoptosis, transfected cells were cultured for 24 h (MGCs) or 48 h (OECs) with 10^−5^ M of corticosterone before apoptosis examination. For MGCs, while percentages of apoptotic cells increased (Figure 5B), the Bcl2/Bax ratio decreased significantly following transfection with Lrp1 siRNA-1 (Figure 5C), compared with cells transfected with negative control siRNA. For OECs, however, transfection with Lrp1 siRNA-3 neither increased percentages of apoptotic cells (Figure 5E) nor decreased the Bcl2/Bax ratio (Figure 5F). Thus, the results suggest that while tPA protected MGCs from corticosterone-induced apoptosis by interacting with LRP1, its protection for OECs did not involve LRP1. 

### 3.5. Effects of Knocking Down the Anxa2 Gene on Corticosterone-Induced Apoptosis of MGCs and OECs

**Figure 6:**
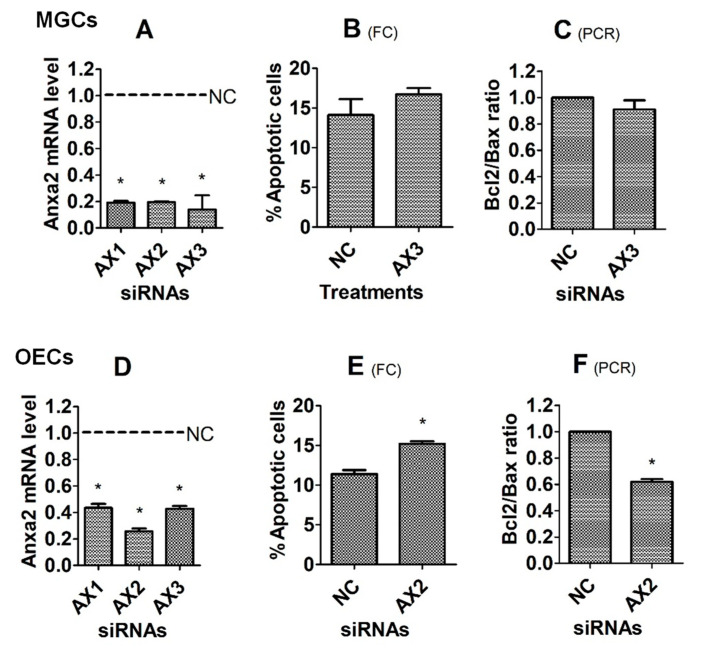
Effects of knocking down Anxa2 on corticosterone-induced apoptosis of MGCs and OECs. Graphs (**A**,**D**), (**B**,**E**) and (**C**,**F**) show Anxa2 mRNA level, percentage of apoptotic cells, and the ratio of Bcl2/Bax mRNA, respectively, after MGCs or OECs were transfected with negative control (NC), Anxa2 siRNA-1 (AX1), -2 (AX2) or -3 (AX3). While the mRNA level was revealed by RT-PCR, the percentage of apoptotic cells was measured by flow cytometry (FC). Each treatment was repeated 3 times, with each replicate including cells from 2 wells of a 6-well plate from 12 mice (MGCs) or from 4 mice (OECs). * Indicates significant difference (*p* < 0.05) from NC cells. In graphs (**B**,**E**), the percentages of apoptotic cells included both early and late apoptotic cells. In graphs (**C**,**F**), the ratio of Bcl2/Bax mRNA in NC cells was set to one, and the ratio in AX3 or AX2 cells was expressed relative to it.

Subsequently to step 3.4, the Anxa2 gene was knocked down by RNA interference before assays for corticosterone-induced apoptosis. To test the silencing efficiency of different siRNA sequences, levels of Anxa2 mRNA were measured by RT-PCR immediately following transfection of MGCs or OECs with negative control, Anxa2 siRNA-1, -2 and -3. While the Anxa2 mRNA level decreased to the lowest in MGCs transfected with Anxa2 siRNA-3 (Figure 6A), it decreased to the lowest in OECs transfected with Anxa2 siRNA-2 (Figure 6D). Thus, Anxa2 siRNA-3 and -2 were used for MGCs and OECs, respectively. To test the effects of gene knocking-down on corticosterone-induced apoptosis, transfected cells were cultured for 24 h (MGCs) or 48 h (OECs) with 10^−5^ M of corticosterone before apoptosis assessment. Transfection of MGCs with Anxa2 siRNA-3 neither increased percentages of apoptotic cells (Figure 5B) nor decreased the Bcl2/Bax ratio (Figure 5C). However, while percentages of apoptotic cells increased (Figure 5E), the Bcl2/Bax ratio decreased significantly following transfection of OECs with Anxa2 siRNA-2 (Figure 5F), compared with cells transfected with negative control siRNA. Thus, the results suggest that tPA protected OECs from corticosterone-induced apoptosis by interacting with Anxa2, whereas its protection for MGCs did not involve Anxa2. 

## 4. Discussion

The current results demonstrate that tPA was antiapoptotic in both MGCs and OECs, and that it protected MGCs and OECs from corticosterone-induced apoptosis by interacting with LRP1 and ANXA2, respectively. Thus, culture with corticosterone significantly increased apoptosis, while decreasing levels of tPA mRNA and protein in both MGCs and OECs. Culture with tPA significantly ameliorated the corticosterone-induced apoptosis of both MGCs and OECs. Furthermore, knocking down Lrp1 significantly increased corticosterone-induced apoptosis of MGCs while having no effect on that of OECs, whereas knocking down Anxa2 significantly promoted corticosterone-induced apoptosis of OECs while having no effect on that of the MGCs. 

The present results showed that culture with corticosterone significantly decreased the levels of tPA mRNA and protein in both MGCs and OECs. Eberhardt et al. [25] observed that treatment of rat mesangial cells with dexamethasone markedly inhibited cAMP-induced tPA expression. Similarly, Kwon et al. [26] reported that treatment of rat primary astrocytes with hydrocortisone led to a significant down-regulation of tPA activity under both normal and inflammatory conditions. However, Wang et al. [12] showed that cortisol treatment increased expression of the Plat gene in primary human amnion epithelial cells. Furthermore, Kathju et al. [27] demonstrated that incubation with dexamethathone and cAMP increased tPA expression synergistically in rat HTC hepatoma cells. Taken together, these data suggest that glucocorticoids regulate tPA expression in various biological systems but in a tissue-specific manner.

The present study showed that culture with tPA significantly reduced the corticosterone-induced apoptosis of both MGCs and OECs. There are several papers reporting that in vitro culture with tPA inhibit apoptosis in different cells. For example, Liang et al. [14] observed that the addition of tPA to a culture-medium partially reversed the appoptotic effect of propofol on developing hippocampal neurons of rats. Hu et al. [13] showed that culture with tPA inhibited staurosporine- or H(2)O(2)-induced apoptosis in a rat interstitial fibroblast cell line (NRK-49F). Furthermore, Liot et al. [28] found that tPA could rescue cultured mouse cortical neurons from serum deprivation-induced apoptosis. However, there are also papers documenting that in vitro culture with tPA facilitate apoptosis. For example, Yoeruek et al. [29] observed that recombinant tPA showed toxic effects on cultured human corneal endothelial cells. Bertrand et al. [30] reported that single-chain tPA could promote N-methyl-D-aspartate receptor-induced calcium influx and subsequent excitotoxicity. Thus, these in vitro studies confirmed the opposite pro- and anti-apoptotic effects of tPA in different cell types. 

This study showed that while tPA protected MGCs from corticosterone-induced apoptosis by interacting with LRP1, it protected OECs from the apoptosis by acting on ANXA2. Although there are some reports that ANXA2 [31,32] and LRP1 [33,34] are involved in regulation of apoptosis, reports on the role of tPA receptors in the tPA regulation of cell apoptosis are very limited. Hu et al. [13] reported that while LRP1 deletion or knockdown abolished the tPA-mediated cell survival, re-introduction of an LRP1 minigene in a mouse LRP-1-deficient fibroblast cell line (PEA-13) restored tPA cytoprotective capability. In contrast, Liot et al. [28] observed that prevention of the interaction between tPA and LRP failed to change the antiapoptotic activity of tPA in cultured mouse cortical neurones. Whether tPA would interact with ANXA2 to prevent apoptosis in the mouse cortical neurones is worth exploring. Taken together, the data from this study provide the first evidence that tPA may use different receptors to inhibit apoptosis in different cell types. 

In summary, although studies suggest that elevation of CRH and/or glucocorticoids associated with female stress-impaired oocyte competence and embryo development, by inducing apoptosis of MGCs and OECs, respectively, the mechanisms for these hormones to induce apoptosis of ovarian and oviductal cells are largely unclear. While tPA has been involved in cell apoptosis, its proapoptotic and antiapoptotic effects have been reported in different studies. This study has explored the role and action mechanism of tPA in corticosterone-induced apoptosis of mouse MGCs and OECs, and the results demonstrate that tPA was antiapoptotic in both MGCs and OECs, and it protected MGCs and OECs from the corticosterone-induced apoptosis by interacting with LRP1 and ANXA2, respectively. The data obtained will provide insights into the mechanisms by which stress hormones damage oocytes and embryos, and may provide new targets for specific inhibition of cancer growth, as it was reported that antibody-directed neutralization of ANXA2 inhibited neo-angiogenesis and human breast tumor growth [35].

## 5. Conclusions

The current results demonstrated that tPA is antiapoptotic in both MGCs and OECs, and that it protects MGCs and OECs from corticosterone-induced apoptosis by interacting with LRP1 and ANXA2, respectively. These data suggest that tPA may use different receptors to inhibit apoptosis in different cell types and will provide insights into the mechanisms by which stress hormones damage oocytes and embryos. Cumulatively, detailed explanation of molecular mechanisms and intracellular networks responsible for inter-transcriptomic and inter-proteomic crosstalk, which are both elicited by pro- and anti-apoptotic pathways initiated in the ex vivo-expanded MGCs and OECs, might be helpful for increasing the usefulness of MGC- and OEC-based culture engineering for the purposes of advanced assisted reproductive technologies (ARTs) and in vitro embryo production (IVP) strategies applicable in mice and other mammalian species. Taking all the aforementioned facts into consideration, different approaches to improvements in the extracorporeal proliferation rates of MGCs and OECs, by simultaneously mitigating the apoptotic events, might provide abundant sources of nuclear donor cells for somatic cell cloning [36,37,38,39] and excellent supplies of feeder monolayers for the systems of MGC- and/or OEC-assisted co-culture with IVP-derived embryos [40,41,42,43].

## Figures and Tables

**Table 1:** Oligonucleotide primer sequences used for real-time PCR in this study.

Gene	Primer Sequences (5′—3′)	Amplified Product Size (bp)
*Plat*	F: ACCGAAAGCTGACGTGGGAA	128
	R: CTGCCAAGGGTGTGAGGTGA	
*Bcl-2*	F: TTCGGGATGGAGTAAACTGG	157
	R: TGGATCCAAGGCTCTAGGTG	
*Bax*	F: TGCAGAGGATGATTGCTGAC	173
	R: GATCAGCTCGGGCACTTTAG	
*Lrp1*	F: GACGAGTGTTCCGTGTATGG	384
	R: CAGGCTGAGGGAGATGTTG	
*Anxa2*	F: CACGAAATCCTGTGCAAGCTC	293
	R: AGGCCCAAAATCACCGTCTC	
*Gapdh*	F: AAACCRGCCAAGTATGATGA	244
	R: GTGGTCCAGGGTTTCTTACT	

## Data Availability

The data presented in this study are available from the corresponding author upon request.

## Abbreviations

ANXA2, Annexin 2; Bax, BCL2 associated X; Bcl-2, B-Cell CLL/Lymphoma 2; LRP1, Low-density lipoprotein receptor-related protein 1; MGCs, Mural granulosa cells; OECs, Oviductal epithelial cells; Plat, Plasminogen activator, tissue type; tPA, Tissue plasminogen activator.
